# Bibliometric Analysis of Global Research Productivity on Vitamin D and Bone Metabolism (2001–2020): Learn from the Past to Plan Future

**DOI:** 10.3390/nu14030542

**Published:** 2022-01-27

**Authors:** Ahmad Azam Malik, Mukhtiar Baig, Nadeem Shafique Butt, Muhammad Imran, Sami Hamdan Alzahrani, Zohair Jamil Gazzaz

**Affiliations:** 1 Department of Family and Community Medicine, Faculty of Medicine in Rabigh, King Abdulaziz University, Jeddah 21589, Saudi Arabia; nshafique@kau.edu.sa; 2 Department of Biochemistry and Medical Education, Faculty of Medicine in Rabigh, King Abdulaziz University, Jeddah 21589, Saudi Arabia; mbbaig@kau.edu.sa; 3 Department of Surgery, Faculty of Medicine in Rabigh, King Abdulaziz University, Jeddah 21589, Saudi Arabia; minmuhammad@kau.edu.sa; 4 Department of Family Medicine, King Abdulaziz University Hospital, Jeddah 21589, Saudi Arabia; salzahrani4@kau.edu.sa; 5 Department of Medicine, Faculty of Medicine in Rabigh, King Abdulaziz University, Jeddah 21589, Saudi Arabia; zjgazzaz@kau.edu.sa

**Keywords:** vitamin D, rickets, bone, bone metabolism, bibliometrics

## Abstract

Vitamin D has long been known for preserving bone and non-skeletal health. Despite its magnitude of impact, scarce literature has explored the evolution of the relevant published literature. This study aimed at evaluating the trends and performance of Vitamin D and bone metabolism-related publications (2001–2020). All pertinent English language 16,887 articles were searched and extracted from the Web of Science (WoS) database and “R-Bibliometrix” was used for comprehensive analysis. Around 60,149 authors contributed from 114 countries, showing the USA on top with >1/4th of all articles, followed by Japan, China, and the United Kingdom. For authors, Cooper C, Dawson-Hughes B, and Holick MF were found to have written the most articles, citations and highest h-index, respectively. Corresponding authors from the top 20 countries collectively were responsible for around 84% of the articles. Among 2735 sources, Osteoporosis International (632), Journal of Bone and Mineral Research (569), and Bone (448) were foremost. Most prominent sources showed recent declining contributions. The increasing trend of publications with a higher spike from 2008 to 2014, followed by a gradual increase till 2017, was observed. Leading countries, affiliations, and authors showed collaborative publications and were mostly from developed countries with limited contributions, particularly from low- and middle-income countries.

## 1. Introduction

Vitamin D (VD) is a lipid-soluble vitamin (a micronutrient) and an essential multi-function bioactive compound for human use [1]. It is not present abundantly in food supplies. It is produced by UV radiation in the skin and is extracted from foods derived from animals (vitamin D3) and plants (vitamin D2) [2,3]. Ultraviolet B (UVB) dermal synthesis exists as the primary means to receiving VD, containing 90% of a VD refill, whereas there are few natural sources of VD, such as various species of fish (salmon, sardines, tuna, mackerel), egg yolk, cod liver oil, organ meat [4]. Nevertheless, the rate of synthesis is impaired by a range of variables, including melanin pigment density; the usage of sunscreen and UV creams and apparel; VD dermal synthesis declines in aging populations due to aging or scarring of the skin; sun-exposure time; the month of the year; and sun-exposure period [5,6,7,8,9]. The VD receptor (VDR), a part of the steroid receptor clan, mediates the biological action of 1,25(OH) 2 D [10]. VDRs are widely distributed in the body. Some fat-related hormones, such as VD, estrogens, and androgens, greatly influence bone metabolism. Additionally, parathyroid hormones, calcitonin, calcium, physical activity, and aging often influence bone metabolism [11,12]. Deficient VD has been addressed as a common and persistent global issue [13]. Low levels trigger rickets and osteomalacia in children and adults, respectively, but these problems are rare in most advanced countries. Still, a subclinical deficiency of VD is more frequent and can be related to osteoporosis and more significant fractures or falls. Deposition of bone minerals starts during pregnancy, particularly during the third quarter [14]. VD is critical for preserving bone health and facilitates in avoidance of non-skeletal illnesses like reproductive, cardiovascular, neoplastic, and metabolic diseases and reproductive disorders [15]. 

VD’s main functions include facilitating calcium and phosphate absorption through intestine synthesis and bone metabolism regulation. VD has an essential role along with PTH in strongly regulating serum ionized calcium concentrations [16]. Repletion of VD is linked with declines in the frequency and severity of many non-musculoskeletal diseases [17], involving diabetes mellitus (T1D and T2D), insulin resistance, and metabolic syndrome [18,19], neurocognitive impairment, certain cancers, depression, infectious diseases, autoimmune diseases, and cardiovascular diseases (CVDs) [16,20,21,22,23]. The epidemiological trials performed with normal, long-term 25(OH)D concentrations have indicated decreased chances for these conditions higher baseline levels of VD. Still, several such observations have not been substantiated by RCTs [16]. While adequate VD is essential for muscles’ proper functioning and skeletal growth and maintenance, data shows that VD enables the prevention of many illnesses, including DM, elevated blood pressure, autoimmune disorders, and some common cancers. As a result, the body’s mechanisms cannot function optimally in the presence of inadequate VD [24]. VD deficiency, as measured by serum 25(OH)D levels of <30 ng/mL, is linked with elevated risks of disease and illness and elevated all-cause mortality even in seemingly healthy people [25,26]. 

Notably, over the last few decades, the scope of usability and the role of VD in health and disease is expanding with increased research output. Its implications among population and researchers across diverse health care specialties and other stakeholders, including policymakers, have been established [13]. Despite its known significance and magnitude of the already conducted research, various VD-related scientific issues still require additional and/or continued exploration and scholarly contributions. Thus, it can be considered essential to assess the research performance and trends in relevant areas to better plan and prepare for future research. Importantly, limited exploration of VD scientific output evaluation has been explored at global [27] or regional levels [28]. However, the relevance of VD with bone metabolism has established its distinct entity. Besides, the literature indicates that research trends and performance analysis related to VD and bone metabolism have not been discussed previously. The current study provides a broad overview of the available scientific literature on this topic and subjective and critical summaries of chosen scientific articles. This study explored the comprehensive research productivity of VD and bone metabolism in the last two decades (2001–2020), using a wide variety of established metrics. Bibliometrics is a recognized analytical entity used in numerous fields to assess research patterns and results [29,30]. Bibliometric studies are techniques for evaluating and characterizing research output and trends. Moreover, it identifies the leading institutions and nations, leading authors and journals, top-cited works, and other important bibliometric indices. Findings can be beneficial to researchers, physicians, veterinarians, legislators and all relevant stakeholders to improve knowledge and standardize information [31,32]. Moreover, exploring all aspects of VD was not considered in the scope of this study, and the current study is limited to a specific area—namely, VD and bone metabolism. The present study aimed to classify and evaluate research publications on VD and bone metabolism to delineate the key contributors in terms of authors, affiliations, countries, and sources. Besides, to expose the main research fields and collaborations present related to VD and bone metabolism-related literature, based on the publication and citation data. 

## 2. Materials and Methods

This scientometric study explored all the published documents on Vitamin D and bone metabolism over the last two decades (2001–2020). Several databases are available with unique advantages and limitations for literature search, such as Web of Science (WoS), Scopus, PubMed, among others. For this study, the required literature search and data extraction were conducted on a Clavirate Analytics database “WoS” [33]. The WoS database (formerly Thomson Reuters) is mostly considered as one of the standardized, consistent [34], and comprehensive, available sources of scientific literature with the highest quality indexing [35]. Its diverse scope, ability to analyze the productivity of various organizations, authors, and countries [36,37] and better compatibility for salient data analysis tools, further add to its usability. Notably, over the last few decades, WoS has been extensively used in various scientometric studies in medicine [27,36,38,39] and other fields [37,40,41,42,43]. 

**Figure 1:**
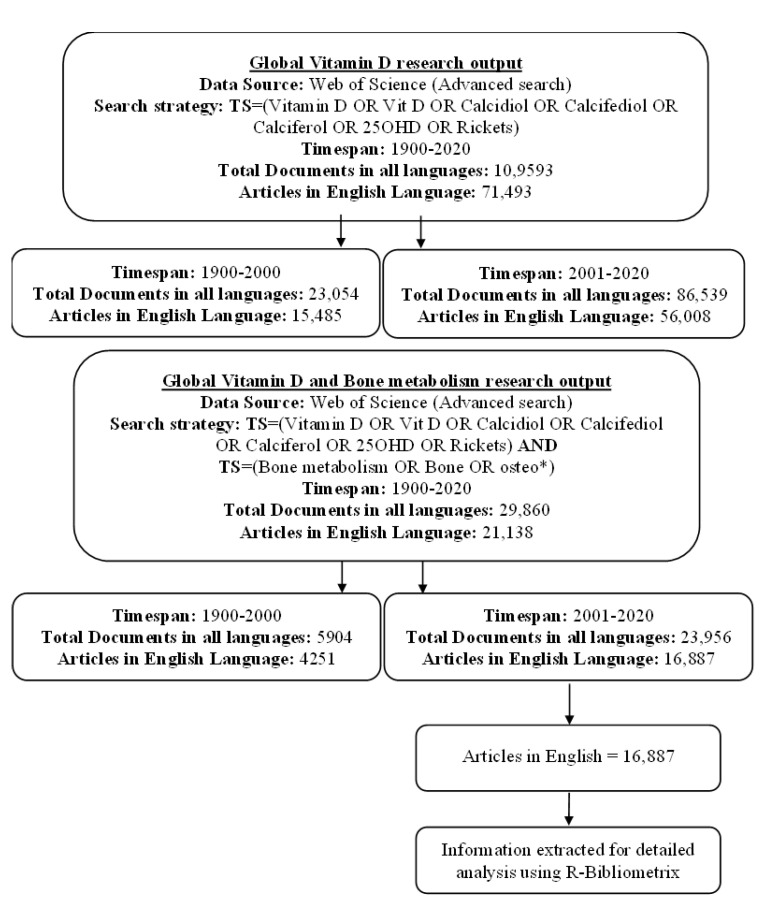
Global Vitamin D and Bone metabolism research output (1900–2020). “*”: Wildcard search in all search fields that allow words and phrases.

King Abdul Aziz University (KAU) online library and digital resources were used to access information. The search strategy was planned after vigilantly selecting the appropriate search terms from literature and having opinions from relevant field experts for its reliability and validity. The topic (TS) field was selected as it comprises the title, abstract, author keywords, and keyword plus terms. Initially, the terms were identified for a) Vitamin D (Vitamin D OR Vit D OR Calcidiol OR Calcifediol OR Calciferol OR 25OHD OR Rickets), as shown in Figure 1 and b) Bone metabolism (Bone metabolism OR Bone OR osteo*). A later search was conducted by merging the abovementioned terms as shown in Figure 1b. In the year 2021, 5437 documents were published for Vitamin D with 3773 articles in English while Vitamin D and Bone metabolism showed 1176 documents with 839 articles. However, documents for the year 2021 were not included or extracted for further detailed analysis. 

A total of 23,956 documents were found to be indexed in WoS (2001–2020) on the topic “Vitamin D and Bone metabolism” (Figure 1a,b). English was the most common language (*n* = 23,153, 96.6%) followed by German (*n* = 229, 1.3%). Articles were the leading type of document with *n* = 17,609 representing 73.5% of all documents followed by reviews (*n* = 3979, 16.6%) and meeting abstracts (*n* = 1579, 6.6%). Some information such as the type of documents, research areas, web of science categories (WC) were extracted using the intrinsic WoS data analyzer function. 

For detailed analysis, all articles in the English Language (*n* = 16,887) were extracted in plain text files from WoS, as shown in Figure 1b. Two researchers (AAM and MB) independently searched and extracted articles to verify the process on the same day (1st October 2021). Later, for detailed analysis, the R-Bibliometrix package [44] was used. Among several tools used in scientometrics to evaluate research performance, R-Bibliometrix is considered to provide comprehensive analysis using an extensive range of indicators at documents, authors, and sources levels. 

## 3. Results

The search strategy yielded a total of 16,887 articles from 2735 sources on the topic of VD and bone metabolism, as shown in Table 1. The total number of authors was 60,149. The majority of the articles were multi-authored, while 4.5% (756) were single-authored articles contributed by <1% (577) of total authors. Open access articles were 44.5%. The collaborative index was 3.69. There were 8241 funding sources, supporting around 47% of total articles. 

**Figure 2:**
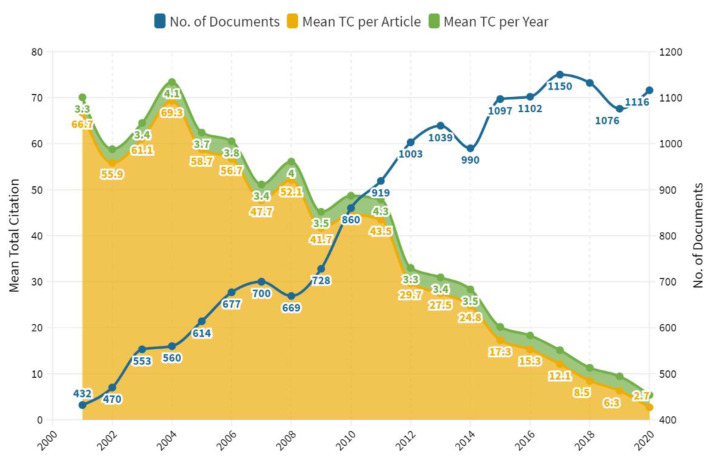
Year-wise publications and total citations (2001–2020). TC—Total citations.

The annual evolution of scientific production is displayed in Figure 2. The number of articles was generally increasing with a maximum in 2017 (1150). A spike with increased production was observed from 2008 to 2013, followed by a gradual increase until 2017. The annual publication growth rate in the last two decades was 4.5%. Higher mean total citations per article and year were observed for the previous decade (2001–2010) with a maximum in 2004.

**Figure 3:**
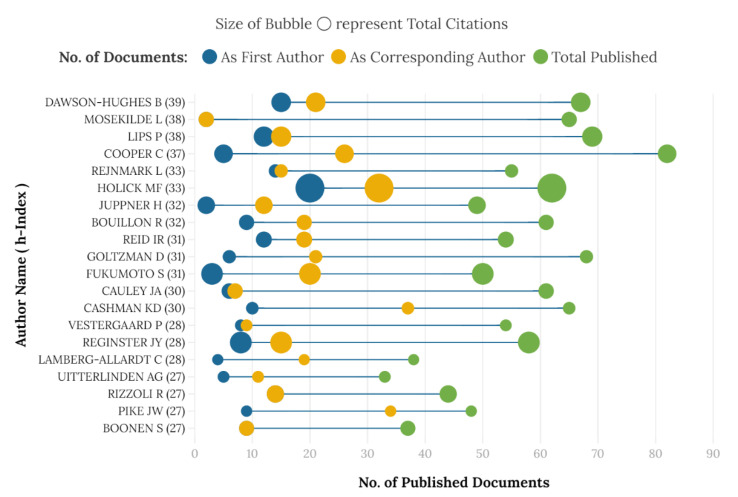
Top 20 most prolific authors and their impact (2001–2020).

Figure 3 shows the top 20 most prolific authors and their impact. Author Cooper C was found to be the most productive with 82 articles. Holick MF had the most citations (15,412) while Dawson-Hughes B showed the highest h-index of 39.

Table 2 shows the top 20 contributing countries in terms of articles and corresponding authors. There were 61,095 country appearances from 115 countries while 94 countries were found to have corresponding authors. Among those, the most productive country was the USA (*n* = 15,838, 26.30% as CA) followed by Japan, China, the United Kingdom, and Italy. Corresponding authors from the top 5, 10, and 20 countries were collectively responsible for 49%, 65.7%, and 85.2% of total articles, respectively. Most of the articles were found to be from single country representation (81%) and led by the USA while Switzerland showed relatively more multiple country publication (MCP) ratio followed by Netherland and the United Kingdom. High Income countries contribution was 81.3% (*n* = 49,693) followed by upper middle income, lower middle income & low income with 13.78% (*n* = 8418), 4.74% (*n* = 2894) & 0.15% (*n* = 90), respectively (table with countries contribution along with their socioeconomic status shared in supplementary files).

This study found that most of the multi-country publications were between authors from two countries. Among those, USA/Canada showed to have maximum (315) collaborations followed by USA/UK, USA/China, USA/Japan, and USA/Germany, with 214, 206, 150, and 143 collaborations, respectively.

**Figure 4:**
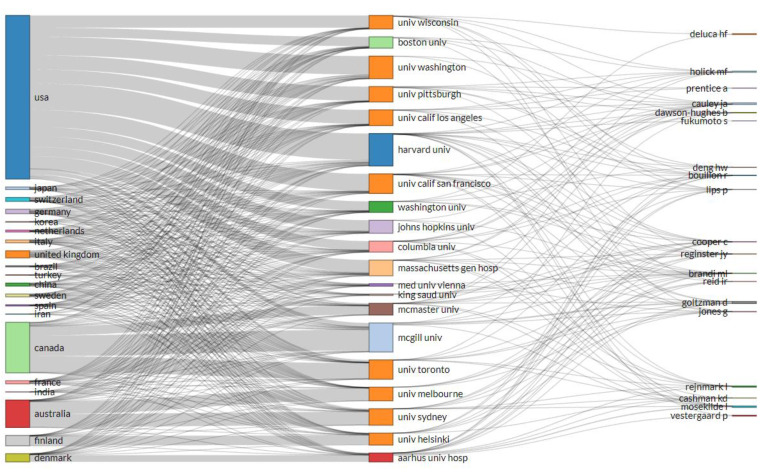
Three Field Plot for top 20 most productive countries, affiliations, and authors.

Table 3 shows the top 10 most frequent affiliations and funding sources. Harvard University and McGill University were leading affiliations. The leading funding organizations were the United States Department of Health Human Services and the National Institutes of Health (NIH). Figure 4 shows a Sankey diagram for the top 20 most productive countries, affiliations, and authors showing dominant contributions by institutes from the USA, followed by Canada and Australia. The Sankey diagram displays how quantities are distributed among items (countries, affiliations, and authors). Thickness of the links (connections) shows high volume of flow between set of values.

Among countries, the USA, Japan, and the United Kingdom showed maximum citations with 208,165, 32,834, and 32,007 citations, respectively. As shown in Table 4, the author Holick MF (USA) was the leading contributor with 5 highly cited articles. In terms of sources, “The Journal of Clinical Endocrinology & Metabolism” and “The American Journal of Clinical Nutrition” were the leading contributors with 4 articles. In terms of study types, mostly were research articles (10, including 4 animal studies) followed by clinical trials and reviews, 4 in each category, respectively. Three (3) and 10 articles showed ≥500 internal (within study selected articles) and ≥1000 global citations, respectively. 

**Figure 5:**
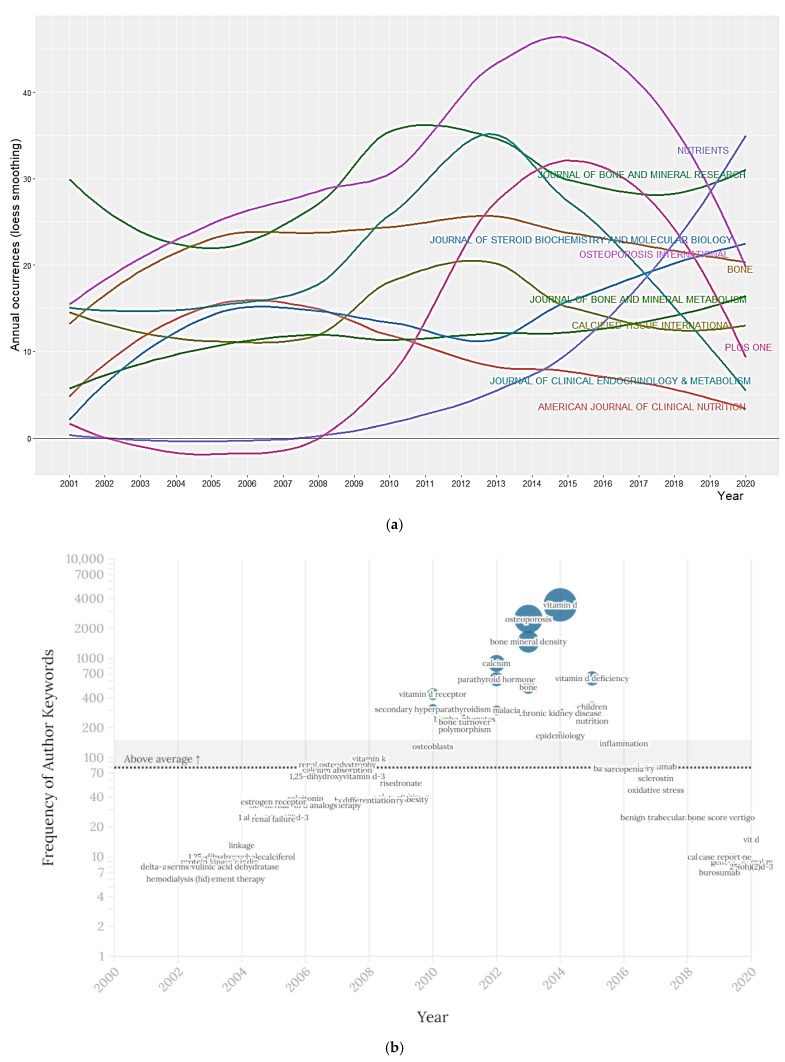
(**a**) Top 10 most productive sources. (**b**) Top 3 keywords per year trends (2001–2020).

Figure 5a shows the year-wise growth of the 10 most productive sources over the last 2 decades. Osteoporosis International, Journal of Bone and Mineral Research, and Bone were the leading sources with 632, 569, and 448 articles, respectively. Among research areas and web of science categories (WC), Endocrinology Metabolism was the leading contributor.

Figure 5b shows the distribution of the most frequent (top 3) keywords trends (2001–2020). In total, 16,775 author keywords were used. The 20 most frequently used keywords occurred from a minimum of 245 to a maximum of 3468 times. Vitamin D (*n* = 3468), osteoporosis (*n* = 2499), bone mineral density (*n* = 1474), calcium (*n* = 891) and vitamin D deficiency (*n* = 636), were the most frequently used keywords. A relatively increased frequency of leading keywords was observed between 2012 to 2015.

## 4. Discussion

Vitamin D is a vitamin cum steroid hormone. It was originally recognized as a biomolecule used in the body for calcium and phosphate homeostasis. However, now VD has multiple non-skeletal preventive and therapeutic applications for maintenance and improvement of health. Thus, extensive research over this prohormone seemed imperative. Quality research work in healthcare generates critical evidence to fill the gaps in our knowledge of health and disease. 

This study found that since the year 1900, >2/3rd of VD-related research articles in English have been published in the last two decades (2001–2020). When explored for VD and bone metabolism, >80% (16,887) of total articles in English were published in the same timeframe. This study summarizes patterns and results over the last two decades (2001–2020) to provide a clearer understanding of the overall productivity of VD and bone metabolism research. Besides, it offers a connection to examine developments in science study on the use of the podium for bibliometrics. Only 1% of articles were single authored with a dominant majority of multi-authored articles in the last 2 decades. The annual growth rate for publications was found to be 4.5%. The trend showed a steady rise in the number of publications with maximum publications in 2017 (1150). Nearly two-thirds of the articles were published over the last 10 years (2011–2020). A spike of maximum productivity was seen from the year 2008 to 2013, producing around 31% of total articles (5218), followed by a gradual increase until 2017. During 2 months in the summer of 2011, more than 500 publications were made on VD mostly about its non-skeletal functions [15]. A similar declining trend of VD and musculoskeletal research output was also observed in a study on VD with co-word analysis [45]. The following decline in publication trend after 2014 could presumably be due to the several factors such as researchers diverted toward other aspects of VD like its role in reproduction [46], diabetes mellitus [19], gestational diabetes [47], CVD and obesity [22], asthma [48], autoimmune diseases [49], depression, hypertension, cancers [50], and many other aspects [45]. 

The retrieved articles were geographically distributed through 114 countries and among those, 32 contributed with ≥100 articles on the study topic. The United States, with 1/4th of the total articles, was the most productive country, followed by Japan, China, and the UK. Study findings point towards major contributions and better scientific output quality from developed countries, primarily the USA, UK, Canada, and European countries. Approximately >3/4 of the published articles related to this topic were published from the western world, especially from the USA. Study findings showed some similarities with other studies conducted on global VD [27] and osteoporosis [51] research productivity showing major contributions by western and developed countries led by the USA but a bit in a different order. Besides the western world, Japan, China, Turkey and Korea also showed decent contributions. Notably, among the top 20 contributing countries, India and Iran were the only 2 from LMICs.

Osteoporosis is the most common bone metabolic problem, and its prevalence is worldwide. In the Eastern Mediterranean Region, the overall pooled osteoporosis prevalence was 24.4% [52]. Research reports from various South and Southeast Asia countries have shown, with few exceptions, a common prevalence of hypovitaminosis D (VD deficiency/insufficiency) in both genders and all age groups of the populace [8]. The Middle East and Africa register the highest rates of rickets worldwide, amid abundant sunlight, and low VD levels are prevalent in the region [53]. VD is synthesized with sunlight. Surprisingly, despite abundant sunlight in several Asian countries, osteoporosis and osteopenia have a high incidence [53,54,55,56]. With the common prevalence of hypovitaminosis D and the high incidence of osteoporosis, one can expect high productivity in research; however, the present study results indicate that the VD and bone metabolism research publication and quality are significantly lower than the western world.

Though, VD deficiency and relevant issues are considered global affecting most parts of the world. It has been reported commonly in the developed world and growing in the Americas and Europe [13,27,57], yet the situation is worst in less developed countries. Study findings also found marginal contributions from many affected regions and low- and middle-income countries. It can also be attributed to another study which found that around 31% of funded articles were also written in the USA and other developed countries. Furthermore, open access articles made up 39.5%, indicating the limitation in terms of access for stakeholders in less developed regions. There could be multiple reasons, such as lack of available resources in terms of funding, unavailability of state-of-the-art research laboratories, lack of qualified and skilled workforce required, fewer quality journals produced from these countries, etc. Most open-access journals charge article processing charges (APC) ranging from 1500–5000 dollars per article. Such a huge APC amount may be one of the hurdles in publishing good quality research in high-impact factor journals. One of the reasons for low research output from middle-income and lower-middle-income countries could be that we conducted a search on the Web of Science only and many journals published from such countries are not indexed on the Web of Science. 

Most of the top 20 prolific authors were from the USA, Europe, UK, and Canada. Among those, 19 authors contributed with ≥50 articles. Authors: Dawson Hughes B (USA), Mosekilde L (Denmark), Lips P (Netherlands), and Cooper C (UK), were found to be the most productive. In terms of impact, one author, Holick MF (USA) had the most citations, followed by Reginster JY (Belgium) and Dawson-Hughes B (USA). Seven (7) authors showed an h-index of ≥30 and were led by Dawson-Hughes B (USA), Lips P (Netherlands), Mosekilde L (Denmark), Cooper C (UK), and Holick MF (USA). Authors: Mosekilde L and Cauley JA had relatively limited numbers of articles as first or corresponding authors. Six (6) authors contributed with >30% articles as corresponding authors led by Deng HW (USA), Deluca HF (USA), and Cashman KD (Ireland), while two authors, Sato Y (Japan) and Holick MF (USA), contributed >20% as first authors. 

These findings suggest that some of the authors are potentially established senior researchers in the field due to their consistent contributions throughout 2 decades, such as Holick MF and Dawson-Hughes B, among others. For corresponding authors, the most productive country was again the USA, with >1/4th of the total articles, followed by Japan, China, the United Kingdom, and Italy. Corresponding authors from the top 20 countries collectively were responsible for 84.3% of the total articles. Interestingly, among these top 20 prolific authors, all had started publication year from 2000 to 2002 in the study timeframe and mostly showed notable productivity with impact. Most of the articles were found to be from single country representation and led by the USA, while Switzerland and Netherlands showed relatively more multiple country publication (MCP) ratio, followed by Canada, Germany, and the United Kingdom. This finding showed a relatively increased trend of multi-country contributions from European countries. Leading countries, affiliations, and authors have produced collaborative publications, mainly from the USA, Europe, Canada, and the UK, with small contributions from low-and middle-income nations. Despite having the highest single country contribution, the USA seemed to be the hub of collaborations and affiliations dominating two of the three clusters found among leading affiliations. Similar trends were observed for countries with maximum total citations. Fourteen institutes contributed with >200 articles, and among them, four (4) showed >300 articles—namely, Harvard University, Mcgill University, University of Wisconsin, and the University of Toronto. Like leading affiliations, the leading funding organizations were also from the USA with two; United States Department of Health Human Services and National Institutes of Health NIH, as the leading funding sources with >2000 contributions. These findings are also aligned with the finding of a relevant study, which is that the USA also had the lead in funding sources and affiliations [27]. Perhaps, it aligns with the higher publication power of the USA, UK, and other resourceful and developed countries with established platforms [27,58].

For the top 20 most cited documents, 12 (60%) documents were published between 2001 and 2006. The top-cited articles cover a diverse range of topics. The author Holick MF (USA) was the leading contributor with 5 highly cited articles (25%), followed by Shimada T (Japan) with 3 articles. In terms of sources, “The Journal of Clinical Endocrinology & Metabolism” was the leading contributor with 4 articles (20%), followed by “The New England Journal of Medicine (NEJM)” and “The American Journal of Clinical Nutrition”. Six (6) and 8 articles showed ≥200 internal (within study selected articles) and ≥1000 global citations, respectively. Holick MF was also the author of the most cited article (>4000 citations), which was published by “The Journal of Clinical Endocrinology & Metabolism” in 2011. 

Among 2617 sources, 14 journals contributed with ≥100 articles in the study timeframe. Most of the top 20 journals were in Q1 and/or Q2 category of JCR quartiles. Journals: Osteoporosis International, Journal of Bone and Mineral Research, and Bone were the leading contributors. Most of the relevant productive journals were consistent contributors over the last two decades with relatively increased productivity from 2008 to 2016, followed by declining contributory trends. Among research areas and web of science categories (WC), Endocrinology Metabolism was the leading contributor, followed by Nutrition Dietetics, Urology Nephrology, Medicine General Internal, and Biochemistry Molecular Biology. A study showed that endocrine and metabolism, musculoskeletal, cancers, and neuropsychological issues were the major contributors to VD research [45]. Mainly similar trends were also observed in a relevant studies study on VD where the shift towards more extra skeletal aspects showed relatively increasing trends compared to the recent decline in bone-related issues [27,45]. A total of 16,775 keywords were used. Vitamin D, osteoporosis, bone mineral density, calcium, and vitamin D deficiency were the most frequently used ones. However, if we analyze the keywords, it is seen that the keywords cover multidisciplinary topics concerning vitamin D and bone metabolism. Besides, a relatively increased frequency of leading keywords between 2008 to 2014 also aligned with the earlier finding of increased productivity around the same period. 

This study had a few limitations such as data was based on the Web of Science database only. Though data standardization might be a hindrance, adding or comparing it with other databases can potentially provide a more comprehensive picture. Secondly, this study included articles in English only that limit the contributions from certain regions. Furthermore, the lack of similar studies on the issue also limits the margin of discussion with comparisons. 

## 5. Conclusions

This study provides a missing analysis of global research trends and performance on VD and bone metabolism and found the last two decades (2001–2020) to be the most productive. Analysis of all articles published in English showed a generally increasing trend with a higher spike from 2008 to 2013. Though most articles were published in the last decade, leading sources showed a recent decline in relevant productivity and suggested the shifting trend towards non-skeletal aspects related to VD. Major contributions were from western and developed countries led by the USA, with marginal contributions from affected regions like the Middle East, South Asia, and other less resourceful settings. Leading authors, affiliations, funding sources, and collaborations were also found to be mainly from the developed regions showing the limitation in collective global research and contributions. These findings from the past not only provide directions for relevant stakeholders, but also require consideration and suggest exploring feasible options in the future to promote research culture and support for settings with limited resources.

## Figures and Tables

**Table 1:** Summary table (2001–2020).

Description	2001–2020
Articles	16,887
Annual growth rate (%)	5.12%
Open access	7531 (44.5%)
Sources (Journals, Books, etc.)	2735
Average years from publication	8.91
Average citations per article	31.45
Average citations per year per doc	2.884
References	265,177
**Article Contents**	
Keywords Plus (ID)	16,302
Author’s Keywords (DE)	16,775
Authors	60,149
Author Appearances	106,300
Authors of single-authored articles	577 (3.4%)
Authors of multi-authored articles	59,572
**Authors Collaboration**	
Single-authored articles	756 (4.5%)
Articles per Author	0.281
Authors per Article	3.56
Co-Authors per Article	6.29
Authors’ countries	114
Group Authors	313
Collaboration Index	3.69
**Other Information**	
Research Areas	112
Web of Science categories	159
Affiliations	10,177
Funding Sources	8241 (48.8%)

**Table 2:** Top 20 countries with articles and corresponding authors.

Country	Total Articles	As CA	Percentage Contribution	SCP	MCP	MCP Ratio	TC
USA	15,838	4418	26.30%	3588	830	0.19	210,348
Japan	3898	1131	6.72%	1030	101	0.09	32,497
China	3647	1084	6.44%	896	188	0.17	13,860
United Kingdom	2978	826	4.91%	596	230	0.28	30,828
Italy	2965	781	4.64%	653	128	0.16	18,389
Canada	2667	627	3.73%	460	167	0.27	22,110
Australia	2289	583	3.46%	453	130	0.22	21,815
Turkey	1805	602	3.58%	577	25	0.04	6733
Germany	1850	502	2.98%	365	137	0.27	17,146
Spain	1963	488	2.90%	408	80	0.16	10,190
France	2005	406	2.41%	301	105	0.26	15,424
India	1083	430	2.56%	384	46	0.11	6307
Korea	1270	372	2.21%	334	38	0.10	5197
Netherlands	1249	322	1.91%	226	96	0.30	14,693
Brazil	1021	331	1.97%	287	44	0.13	4654
Denmark	990	264	1.57%	212	52	0.20	9900
Iran	916	280	1.66%	257	23	0.08	3836
SWEDEN	950	229	1.36%	168	61	0.27	7052
SWITZERLAND	936	221	1.31%	117	104	0.47	8206
FINLAND	1004	207	1.23%	162	45	0.22	8114

CA—Corresponding author, SCP—Single or Intra-country publication, MCP—Multiple or Inter-country publications, TC—Total citations

**Table 3:** Top 10 most frequent affiliations and funding sources.

Top 10 Most Frequent Affiliations	Articles
Harvard Univ	529
Mcgill Univ	498
Univ Wisconsin	339
Univ Toronto	311
Univ Sydney	301
Univ Calif San Francisco	292
Univ Calif Los Angeles	280
Massachusetts Gen Hosp	256
Univ Melbourne	239
Univ Pittsburgh	239
Top 10 most frequent funding Organizations	Articles
United States Department of Health Human Services	2526
National Institutes of Health NIH USA	2509
NIH National Institute of Diabetes Digestive Kidney Diseases NIDDK	875
NIH National Institute of Arthritis Musculoskeletal Skin Diseases NIAMS	626
National Natural Science Foundation of China NSFC	599
NIH National Center for Research Resources NCRR	525
NIH National Institute on Aging NIA	403
AMGEN	391
Medical Research Council UK MRC	313
Ministry of Education Culture Sports Science and Technology Japan MEXT	303

**Table 4:** Top 20 highly cited Articles.

Title	First Author	Study Type	Source	IF	Year	IC	GC
Evaluation, Treatment, and Prevention of Vitamin D Deficiency: An Endocrine Society Clinical Practice Guideline	Michael F. Holick	Clinical guidelines	The Journal of Clinical Endocrinology & Metabolism	5.958	2011	878	4714
Sunlight and vitamin D for bone health and prevention of autoimmune diseases, cancers, and cardiovascular disease	Michael F Holick	Review article	The American Journal of Clinical Nutrition	7.047	2004	508	1643
Vitamin D: importance in the prevention of cancers, type 1 diabetes, heart disease, and osteoporosis	Michael F Holick	Review article	The American Journal of Clinical Nutrition	7.047	2004	508	1119
Calcium plus Vitamin D Supplementation and the Risk of Fractures	Rebecca D. Jackson	Clinical Trial	The New England Journal of Medicine	91.253	2006	361	1177
FGF-23 is a potent regulator of vitamin D metabolism and phosphate homeostasis	Takashi Shimada	Research article (Animal study)	The Journal of Bone and Mineral Research	6.741	2004	345	1134
Cloning and characterization of FGF23 as a causative factor of tumor-induced osteomalacia	Takashi Shimada	Research article (Animal study)	Proceedings of the National Academy of Sciences (PNAS)	11.205	2001	329	986
Targeted ablation of Fgf23 demonstrates an essential physiological role of FGF23 in phosphate and vitamin D metabolism	Takashi Shimada	Research article (Animal study)	The Journal of Clinical Investigation	14.808	2004	289	1050
Positive association between 25-hydroxy vitamin D levels and bone mineral density: a population-based study of younger and older adults	Heike A Bischoff-Ferrari	Research article	The American Journal of Medicine	4.965	2004	287	539
A Global Study of Vitamin D Status and Parathyroid Function in Postmenopausal Women with Osteoporosis: Baseline Data from the Multiple Outcomes of Raloxifene Evaluation Clinical Trial	Paul Lips	Research article	The Journal of Clinical Endocrinology & Metabolism	5.958	2001	283	534
Use of calcium or calcium in combination with vitamin D supplementation to prevent fractures and bone loss in people aged 50 years and older: a meta-analysis	Benjamin MP Tang	Meta-analysis	The Lancet	79.323	2007	279	824
Effect of Parathyroid Hormone (1–34) on Fractures and Bone Mineral Density in Postmenopausal Women with Osteoporosis	Robert M. Neer	Research article	The New England Journal of Medicine	91.253	2001	275	3022
Prevalence of Vitamin D Inadequacy among Postmenopausal North American Women Receiving Osteoporosis Therapy	Michael F. Holick	Research article	The Journal of Clinical Endocrinology & Metabolism	5.958	2005	266	609
Vitamin D deficiency: a worldwide problem with health consequences	Michael F. Holick	Review article	The American Journal of Clinical Nutrition	7.047	2008	263	1467
Overview of general physiologic features and functions of vitamin D	Hector F DeLuca	Review article	The American Journal of Clinical Nutrition	7.047	2004	257	1312
Effect of four monthly oral vitamin D3 (cholecalciferol) supplementation on fractures and mortality in men and women living in the community: Randomised double blind controlled trial	Daksha P Trivedi	Clinical Trial	The BMJ	39.89	2003	255	843
Fibroblast Growth Factor 23 in Oncogenic Osteomalacia and X-Linked Hypophosphatemia	Kenneth B. Jonsson	Research article	The New England Journal of Medicine	91.253	2003	229	617
Annual High-Dose Oral Vitamin D and Falls and Fractures in Older Women A Randomized Controlled Trial	Kerrie M. Sanders	Clinical Trial	JAMA	56.274	2010	227	829
Klotho converts canonical FGF receptor into a specific receptor for FGF23	Itaru Urakawa	Research article (Animal study)	Nature	49.962	2006	218	1219
Increased Circulatory Level of Biologically Active Full-Length FGF-23 in Patients with Hypophosphatemic Rickets/Osteomalacia	Yuji Yamazaki	Research article	The Journal of Clinical Endocrinology & Metabolism	5.958	2002	213	502
Effects of Vitamin D and Calcium Supplementation on Falls: A Randomized Controlled Trial	Heike A Bischoff	Clinical Trial	The Journal of Bone and Mineral Research	6.741	2003	210	643

IF—Impact Factor, IC—Internal Citation (Citations within study selected documents), GC—Global Citation (Citation in Web of Science).

## Data Availability

The data file used for this study will be shared or uploaded in supplementary files when required and further inquiries can be directed to the corresponding author.

## Supplementary Materials

The following supporting information can be downloaded at: https://www.mdpi.com/article/10.3390/nu14030542/s1, Table S1: Countries contribution along with their socioeconomic status.
